# Improving precision management of anxiety disorders: a Mendelian randomization study targeting specific gut microbiota and associated metabolites

**DOI:** 10.3389/fmicb.2024.1380912

**Published:** 2024-04-09

**Authors:** Ming-Min Xu, Wen-Hui Qiu, Qing-Yu Ma, Zhi-Yun Yu, Wen-Miao Yang, Tian-Nuo Hu, Yu Guo, Xiao-Yin Chen

**Affiliations:** ^1^School of Traditional Chinese Medicine, Jinan University, Guangzhou, China; ^2^Guangzhou Key Laboratory of Formula-Pattern of Traditional Chinese Medicine, School of Traditional Chinese Medicine, Jinan University, Guangzhou, China

**Keywords:** gut microbiota and associated metabolites, anxiety disorders, Mendelian randomization, causal effects, management approaches

## Abstract

**Background:**

There is growing evidence of associations between the gut microbiota and anxiety disorders, where changes in gut microbiotas may affect brain function and behavior via the microbiota-gut-brain axis. However, population-level studies offering a higher level of evidence for causality are lacking. Our aim was to investigate the specific gut microbiota and associated metabolites that are closely related to anxiety disorders to provide mechanistic insights and novel management perspectives for anxiety disorders.

**Method:**

This study used summary-level data from publicly available Genome-Wide Association Studies (GWAS) for 119 bacterial genera and the phenotype “All anxiety disorders” to reveal the causal effects of gut microbiota on anxiety disorders and identify specific bacterial genera associated with anxiety disorders. A two-sample, bidirectional Mendelian randomization (MR) design was deployed, followed by comprehensive sensitivity analyses to validate the robustness of results. We further conducted multivariable MR (MVMR) analysis to investigate the potential impact of neurotransmitter-associated metabolites, bacteria-associated dietary patterns, drug use or alcohol consumption, and lifestyle factors such as smoking and physical activity on the observed associations.

**Results:**

Bidirectional MR analysis identified three bacterial genera causally related to anxiety disorders: the genus *Eubacterium nodatum group* and genus *Ruminococcaceae UCG011* were protective, while the genus *Ruminococcaceae UCG011* was associated with an increased risk of anxiety disorders. Further MVMR suggested that a metabolite-dependent mechanism, primarily driven by tryptophan, tyrosine, phenylalanine, glycine and cortisol, which is consistent with previous research findings, probably played a significant role in mediating the effects of these bacterial genera to anxiety disorders. Furthermore, modifying dietary pattern such as salt, sugar and processed meat intake, and adjusting smoking state and physical activity levels, appears to be the effective approaches for targeting specific gut microbiota to manage anxiety disorders.

**Conclusion:**

Our findings offer potential avenues for developing precise and effective management approaches for anxiety disorders by targeting specific gut microbiota and associated metabolites.

## 1 Introduction

Anxiety disorders are the most prevalent mental disorders, and they usually develop during early adulthood (Penninx et al., 2021). A recent population-based study of 275,057 adolescents aged 12–17 years from 82 countries representing the full spectrum of economic wealth across the six World Health Organization regions reported a 9% 12-month pooled prevalence of anxiety disorders (Biswas et al., 2020). Across all age groups in the US, anxiety disorders have a lifetime prevalence of approximately 34% (Szuhany and Simon, 2022). In creating an environment where many individuals experienced heightened levels of anxiety and stress, the recent COVID-19 pandemic also further exacerbated mental health issues globally, producing an additional 76.2 million cases of anxiety disorders worldwide, representing a 25.6% increase in pre-pandemic levels (COVID-19 Mental Disorders Collaborators, 2021). Individuals with anxiety disorders face an increased likelihood of developing cardiovascular diseases, respiratory disorders, gastrointestinal problems, and other chronic conditions (Penninx et al., 2021; Szuhany and Simon, 2022). They are also at increased risk of depression, substance use disorders, suicidal ideation, and impaired cognitive functioning, including memory, attention, and decision-making difficulties (Penninx et al., 2021; Szuhany and Simon, 2022). Thus, anxiety disorders have emerged as a significant health concern, contributing significantly to the global disease burden (COVID-19 Mental Disorders Collaborators, 2021).

First-line therapies for anxiety disorders, including medication and cognitive behavioral therapy (CBT), have limitations. Medications may cause side effects, and long-term use can result in tolerance and dependence (Penninx et al., 2021; Szuhany and Simon, 2022). CBT requires a significant time and energy commitment, can be costly to administer, and may not be suitable for all patients, often necessitating combination with other therapies (Penninx et al., 2021; Szuhany and Simon, 2022). Anxiety disorders are multifactorial, with both psychosomatic and genetic components contributing to the underlying pathogenesis (Penninx et al., 2021; Szuhany and Simon, 2022), and the precise mechanisms underlying these disorders are complex and still not completely understood. Advances in genetics, particularly genome-wide association studies (GWAS), have improved our understanding of the causes of mental disorders (Meier et al., 2019; Koskinen and Hovatta, 2023). By investigating the complex interplay between genetic and environmental factors, we can gain new insights into the biological mechanisms driving anxiety disorders to inform the development of more effective and personalized therapies.

The gut microbiome is a vast and intricate collection of microorganisms that include bacteria, fungi, viruses, and other microbes residing in the gastrointestinal tract. This microbial community is one of the most extensive and complex communities in the human body; in fact, the microbiome usually contains significantly more cells than the human body (Li et al., 2016). The gut microbiota plays a crucial role in many biological processes including breaking down nutrients, synthesizing vitamins and essential molecules, and maintaining the integrity and stability of the intestinal barrier (Li et al., 2016). Moreover, the gut microbiota interacts with various organs and body systems, including the immune, digestive, nervous, and endocrine systems, significantly impacting human health and influencing essential physiological processes (Li et al., 2016). There is now an extensive body of evidence showing that changes in gut microbiota composition are closely related to various diseases, including metabolic disorders (Agus et al., 2021; Fan and Pedersen, 2021), inflammatory conditions (Virtue et al., 2019; Cai et al., 2022), and mental health disorders such as anxiety disorders (Nikolova et al., 2021; Simpson et al., 2021). The microbiota-gut-brain axis, which describes a pathway through which changes to the gut microbiota affect brain function and behavior, has attracted significant attention (Cryan et al., 2019; Deng et al., 2021; Socała et al., 2021). Several studies have identified a potential connection between the gut microbiota and anxiety disorders, showing that individuals with anxiety disorders exhibit distinct changes in gut microbiota composition compared with healthy individuals including reduced microbial diversity, altered bacterial taxa abundance, and impaired microbial metabolic functions (Tran et al., 2019; Lee et al., 2021; Zhu et al., 2023). In addition, communication between the gut microbiota and brain involves the autonomic nervous system and its corresponding neurotransmitters (e.g., γ-aminobutyric acid (GABA), dopamine) (Dan et al., 2020; Prochazkova et al., 2021), bacterial metabolites such as short-chain fatty acids (SCFAs) (De Vadder et al., 2014; Wang et al., 2020), and amino acid metabolites (Sun et al., 2022; Zhang et al., 2023). Metabolic derivatives also play an important role in providing key neurotransmitters, so relevant metabolites such as tryptophan, the raw material for serotonin, also known as 5-hydroxytryptamine (5-HT), could potentially link the gut microbiota to anxiety disorders (Deng et al., 2021; Tian et al., 2023). On the other hand, there is evidence that the relationship between the gut microbiota and anxiety disorders can be influenced by several confounding factors such as dietary patterns, drug or alcohol use, and lifestyle factors such as smoking and physical activity (Zhu et al., 2020; Simpson et al., 2021; Cataldi et al., 2022). Controlling these factors when investigating complex biological relationships can be challenging, limiting the potential to infer definitive associations between a biological exposure (the gut microbiota) and a clinical phenotype or disease outcome (anxiety disorders). Hence, rigorous studies that utilize controlled methodologies and that isolate these confounding variables are needed.

Mendelian randomization (MR) methods use genetic instruments, normally single nucleotide polymorphisms (SNPs), as instrumental variables (IVs) for modeling and inferring causal effects and potential associations between exposure and disease outcome. MR exploits the fact that the allocation of genotypes from parent to offspring is random, such that the association between genetic variants and outcome is not affected by common confounding factors. Also, because heredity is irreversible, the interference of reverse causation can be excluded (Davey Smith and Hemani, 2014; Burgess et al., 2015). Thanks to large-scale GWAS of gut microbiotas in different diseases, MR has been widely used to study disease pathogenesis. Indeed, several studies using MR have now established that the gut microbiota is strongly associated with several neurological diseases including Alzheimer’s disease (Zeng et al., 2023), major depressive disorder (Chen M. et al., 2022), schizophrenia (Cheng et al., 2023), and insomnia (Li et al., 2023).

In this study, using the GWAS summary statistics, MR analysis was conducted to comprehensively explore whether the gut microbiota has a causal effect on anxiety disorders. We aimed to identify specific bacterial genera linked to anxiety disorders and then investigate the potential impact of various neurotransmitter-associated metabolites, dietary patterns, drugs and alcohol use, and lifestyle factors on these associations. Understanding these relationships has implications for understanding the specific mechanisms driving anxiety disorders and developing sustainable and effective management approaches targeting the gut microbiota and associated metabolites in this common condition.

## 2 Materials and methods

### 2.1 Study design

**Figure 1:**
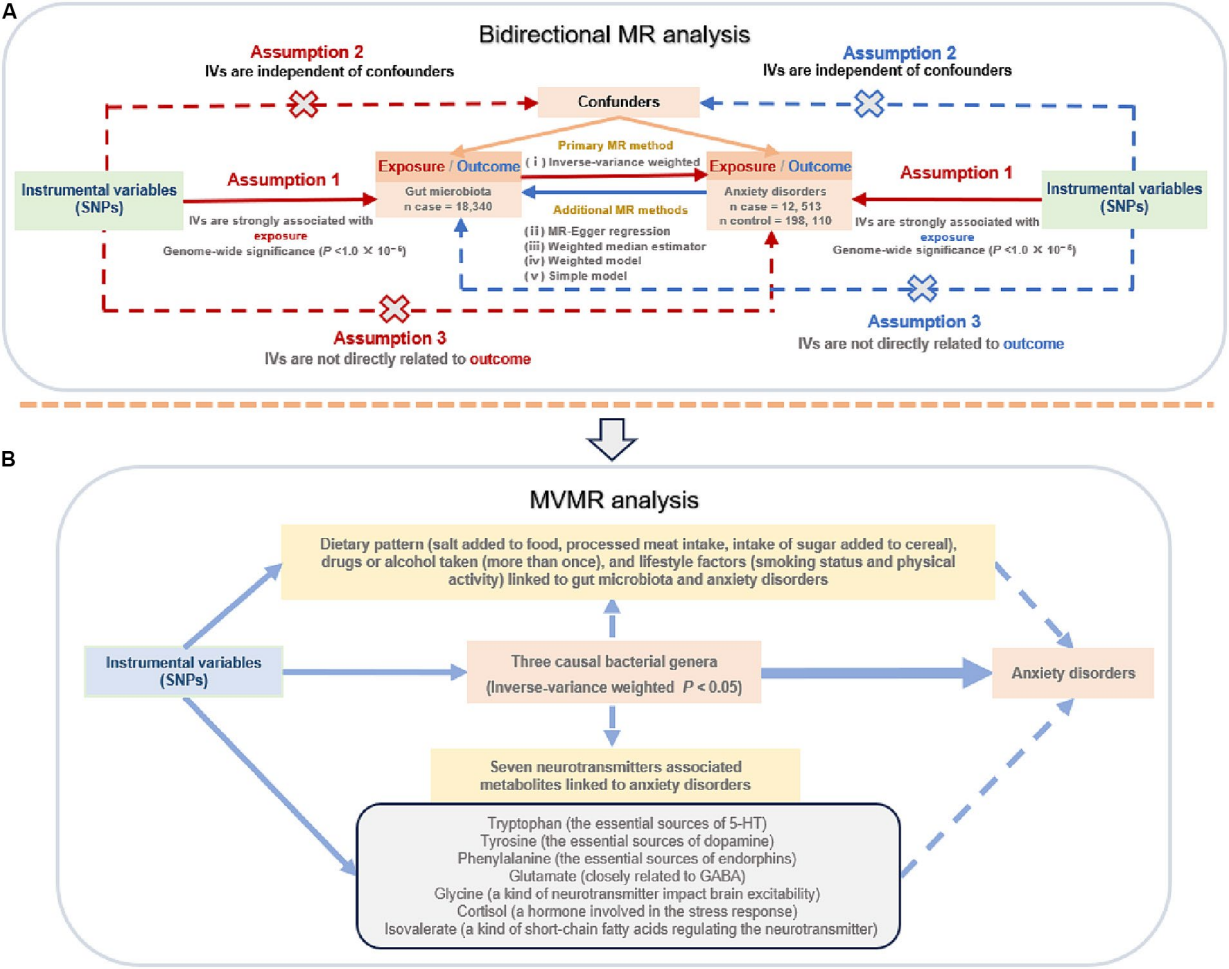
The flow chart of the study design. **(A)** Two-sample, bidirectional Mendelian randomization (MR) analysis; **(B)** multivariable MR (MVMR) analysis. SNPs: single nucleotide polymorphisms; IVs, instrumental variables; 5-HT, hydroxytryptamine; GABA: γ-aminobutyric acid.

The overall flow chart of our study design is shown in Figure 1. To uncover potential associations between gut microbiotas and anxiety disorders, as well as identify specific bacterial taxa at the genus level linked to anxiety disorders, we conducted a two-sample, bidirectional MR analysis. This analysis incorporated several methods and sensitivity analyses of publicly available summary-level GWAS data. Subsequently, we conducted multivariable MR (MVMR) analysis to investigate the potential impact of various common neurotransmitter-associated metabolites linked to anxiety disorders on these taxa-level associations to differentiate between the direct and indirect effects of gut microbiotas associated with anxiety disorders. This approach aimed to identify bacterial genera-associated metabolites that could potentially affect the risk of anxiety disorders. Moreover, MVMR analysis was employed to further explore the potential impact of bacteria-associated dietary patterns, drug and alcohol use, and lifestyle factors (smoking and physical activity) on the causal effects of the identified bacterial genera on anxiety disorders. In performing this comprehensive analysis, we aimed to provide new mechanistic insights and management perspectives for anxiety disorders.

The study was designed and reported according to the Strengthening the Reporting of Observational Studies in Epidemiology—Mendelian Randomization (STROBE-MR) statement (Skrivankova et al., 2021a,b).

### 2.2 Ethics statement

This study used only publicly available GWAS data and published studies that had already undergone ethical review and received participant consent. As such, no additional ethical approval was needed.

### 2.3 Data sources

GWAS summary data for gut microbiotas were derived from the largest, multi-ethnic, genome-wide meta-analysis published to date on gut microbiota composition conducted by the MiBioGen consortium. This study collected whole-genome genotyping data from 18,340 individuals across 24 separate cohorts together with 16S rRNA genotyping of the participants’ fecal microbiomes. Among the 24 cohorts, 20 included samples from individuals of single ancestry, with the majority of participants being of European ancestry (16 cohorts, *n* = 13,266) (Kurilshikov et al., 2021). Three different variable regions (V1-V2, V3-V4, and V4) of the 16S rRNA genotypes were used to profile intestinal microbial species composition, using microbiota quantitative trait loci (mbQTL) mapping to reveal genetic variants governing the abundance of bacterial taxa within the gut microbiota. Notably, the analysis revealed that these variations impacted the relative abundances of nine phyla, 16 classes, 20 orders, 35 families, and 131 genera encompassing 12 unknown genera (Kurilshikov et al., 2021). Finally, 119 genus-level taxa were included in the current study for MR analysis.

GWAS summary-level statistics for anxiety disorders were obtained from the FinnGen consortium R5 release data. The FinnGen project is an academic-industrial collaboration aiming to discover genotype–phenotype relationships in over 500,000 Finnish participants. The phenotype “All anxiety disorders” (description: “a category of psychiatric disorders characterized by anxious feelings or fear often accompanied by physical symptoms associated with anxiety”) and GWAS ID “finn-b-F5_ALLANXIOUS” were adopted in the current study. This GWAS included 210,623 male and female subjects and consisted of 12,513 European cases and 198,110 healthy controls. They collected as many anxiety disorders as possible, resulting in 19 reported traits (e.g., anxiety and stress-related disorders, anxiety disorder, lifetime anxiety disorder, and ICD10 F40 F41 F42: any anxiety disorder) and 11 child traits (e.g., generalized anxiety disorder, social anxiety disorder, mixed anxiety and depressive disorder, acute stress reaction). More information on the study can be found at https://r5.risteys.finngen.fi/.

We also aimed to investigate the potential involvement of neurotransmitter-associated metabolites in the biological pathway(s) linking gut microbiotas to anxiety disorders. Through a systematic literature search and considering commonly measured serum metabolites in metabolomic studies, we identified several significant neurotransmitter-associated metabolites including tryptophan (Deng et al., 2021; Jia et al., 2024), tyrosine (Needham et al., 2022; Zhang D. et al., 2022), phenylalanine (Deng et al., 2022; Zhang D.-D. et al., 2022), glutamate (Zhao et al., 2020; Laroute et al., 2022), and glycine (Lin et al., 2021; Zhang D. et al., 2022). These metabolites have been found to closely associate with the gut-brain axis, and their dysregulation may contribute to the development of anxiety disorders. Furthermore, cortisol is often investigated alongside neurotransmitter-associated metabolites as a potential biomarker or factor in anxiety disorders (Dalile et al., 2020; Tian et al., 2023). In addition, a critical group of bacterial metabolites called short-chain fatty acids, such as isovalerate, has been found to be closely associated with anxiety disorders and was also taken into consideration in our analysis (Bayrer et al., 2023; Raman et al., 2023). We extracted genetic data for these specific human blood metabolites (i.e., tryptophan, tyrosine, phenylalanine, glutamate, glycine, cortisol, isovalerate) from the metabolomics GWAS server.^1^ The most comprehensive analysis to date on the genetic loci associated with blood metabolites was of a sample of 7,824 European participants, which identified almost 2.1 million SNPs for 486 metabolites associated with human genetic variants by genome-wide association scans with high-throughput metabolic profiling (Kanehisa et al., 2012; Shin et al., 2014). Specifically, tryptophan, tyrosine, phenylalanine, and glutamate were directly associated with neurotransmitters, namely 5-HT (Deng et al., 2021; Jia et al., 2024), dopamine (Needham et al., 2022; Zhang D. et al., 2022), endorphins (Deng et al., 2022; Zhang D.-D. et al., 2022), and GABA (Zhao et al., 2020; Laroute et al., 2022). Glycine itself is a neurotransmitter (Lin et al., 2021; Zhang D. et al., 2022). Cortisol, a hormone involved in the stress response, exerts an influence on neurotransmitter activity (Dalile et al., 2020; Tian et al., 2023). Isovalerate, on the other hand, is a short-chain fatty acid that contributes to regulating neurotransmitter synthesis and release, blood–brain barrier permeability, and neuroinflammation (Bayrer et al., 2023; Raman et al., 2023).

Additionally, based on our extensive literature search, we identified potential confounding factors such as diet and lifestyle that might significantly impact both the gut microbiota and anxiety disorders (Zhu et al., 2020; Simpson et al., 2021; Cataldi et al., 2022). These factors include dietary patterns (such as salt added to food, processed meat intake, and intake of sugar added to cereal); drug and alcohol use; and lifestyle factors such as smoking status and physical activity. We therefore conducted further analysis to explore how these factors may influence the causal association between the gut microbiota and anxiety disorders. The GWAS summary statistics for dietary pattern and drug or alcohol use (more than once) were obtained from the UK Biobank release data. Lifestyle factors such as smoking and physical activity were obtained from the European Bioinformatics Institute release data. An overview of the sources of selected neurotransmitter-associated metabolites and dietary pattern, drug or alcohol use, and lifestyle factors related to the gut microbiota and anxiety disorders are shown in Supplementary Table S1.

### 2.4 IVs selection

As shown in Figure 1, SNPs had to meet three core assumptions for inclusion as IVs in our study: (i) correlation—the relationship between genetic variants and exposure was robust; (ii) independence—the genetic variants were independent of any confounding factors affecting exposure and outcome; and (iii) exclusion—the genetic variants influenced the risk of the outcome through exposure rather than other potential pathways (Davey Smith and Hemani, 2014; Burgess et al., 2015).

To ensure the selection of suitable SNPs as IVs from the GWAS statistics, we applied a series of quality control steps. (1) To increase the number of available genetic instruments, consistent with previous studies (Ni et al., 2021; Li et al., 2023; Yu et al., 2023), SNPs at a threshold of *p* < 1.0 × 10^−5^ from GWAS summary data on the exposure, such as the gut microbiota, were selected as potential IVs. (2) To avoid the effect of linkage disequilibrium (LD) between SNPs, European sample data from the 1,000 Genomes Project was used as the reference panel, and the SNPs within a clumping window size of 10,000 kb were pruned under the threshold of *R*^2^ < 0.001 to mitigate LD, thus ensuring independence of each IV. (3) The SNP information associated with exposure was extracted, and palindromic SNPs (e.g., those with A/T or G/C alleles) as well as ambiguous and duplicated SNPs were eliminated after harmonizing the exposure and outcome SNPs for accurate dataset matching. (4) The remaining SNPs with positive results were searched using PhenoScanner^2^ to determine whether there were potential confounders. (5) To assess the extent of weak instrument bias, we computed the F-statistic of the IVs. This statistical measure indicates the strength of the association between the IVs and the exposure. Specifically, an *F*-statistic ≥10 indicates no compelling evidence of weak instrument bias. We excluded IVs with *F*-statistics <10, which suggests that they were weak IVs. The formula for calculating the *F*-value is: *F* = [(*n*−*k*−1) / *k*] × [*R*^2^ / (1−*R*^2^)], where *R*^2^ represents the proportion of exposure variance explained by the IVs, *n* is the sample size, and *k* is the number of IVs (Burgess and Thompson, 2011).

### 2.5 Statistical methods

#### 2.5.1 Two-sample and bidirectional MR analysis

The inverse variance-weighted average method (IVW), the most efficient causal estimation method allowing balanced pleiotropy in MR analysis, was used as the primary analytical method for estimating causal effects of the exposures on the outcome. The IVW method assumes that all SNPs are valid instruments, and the overall effect is estimated by calculating the inverse variance-weighted mean of the effect estimates for the instrumental variables. When there is no horizontal pleiotropy bias present, the IVW method is more statistically powerful (Burgess et al., 2013). In addition to the IVW method, four additional MR methods [MR-Egger regression, weighted median estimator (WME), weighted model, simple model] served as complementary analyses to assess causal associations. The MR-Egger regression method provides unbiased estimates even in the presence of horizontal pleiotropy (Burgess and Thompson, 2017). The WME method can adjust for the effect of invalid IVs. Assuming the presence of up to half of invalid IVs, the WME method yields robust estimates (Bowden et al., 2016). When the largest number of similar individual SNP causal effect estimates are from efficient SNPs, the weighted model was consistent even if SNPs were invalid (Burgess et al., 2013). The simple mode was an unweighted mode of the empirical density function of causal estimation (Burgess et al., 2013).

To ensure the reliability of our results and eliminate interference from reverse causality, we also performed reverse MR analysis. In this analysis, we considered anxiety disorders as the “exposure” and identified bacterial genera that were causally associated with anxiety disorders as the “outcome.” The methods and settings were the same as those used in forward MR.

A series of sensitivity analyses were then performed to examine the consistency of the causal association. First, Cochran’s Q statistic was used to assess heterogeneity, and a *p*-value of <0.05 was considered to indicate the presence of significant heterogeneity in the SNP effect estimates. Second, we employed the MR-Egger intercept test and Mendelian Randomization Pleiotropy RESidual Sum and Outlier (MR-PRESSO) test to detect horizontal pleiotropy (Verbanck et al., 2018). The MR-Egger intercept test compares the MR-Egger intercept term with a value of zero, where a considerable deviation from zero suggests the presence of substantial horizontal pleiotropy. MR-PRESSO evaluates the sum of residuals from each SNP to assess the magnitude of horizontal pleiotropy, and the results of the main analysis method, IVW, can be obtained after adjusting for horizontal pleiotropy. The MR-PRESSO global test assesses the overall horizontal pleiotropy of the IVs and the MR-PRESSO outlier test assesses abnormal SNPs that lead to the existence of overall horizontal pleiotropy. The MR-Egger intercept test and MR-PRESSO global test helped to identify any potential confounding effects due to pleiotropic pathways. If the *p*-value of these tests was >0.05, horizontal pleiotropy was ruled out (Verbanck et al., 2018). Furthermore, we conducted a leave-one-out sensitivity analysis, progressively eliminating each individual SNP to assess the impact of potential anomalies on the overall causal effect.

#### 2.5.2 MVMR analysis

MVMR is an extension of MR that allows SNPs to be associated with multiple exposures, provided that they are not linked to confounding variables of any exposure-outcome associations and have no direct impact on the outcome (Davey Smith and Hemani, 2014; Burgess et al., 2015). In our analysis, we accounted for relevant neurotransmitter-associated metabolites, dietary patterns, drugs and alcohol consumption, and lifestyle factors separately. Using MVMR analyses, including MVMR-IVW and MVMR-Egger, we estimated the independent and direct causal effects of identified bacterial genera on anxiety disorders (Davey Smith and Hemani, 2014; Burgess et al., 2015). This allowed us to explore potential metabolic pathways associated with neurotransmitter-associated metabolites closely linked to the gut microbiota and anxiety disorders. We also examined the potential impact of relevant dietary patterns, drugs and alcohol consumption, and lifestyle factors on the causal effects of the identified bacterial genera on anxiety disorders. Heterogeneity was assessed using Cochran’s Q statistic. The parameter settings adopted for these analyses were the same as those used in the univariate MR analysis and were selected to ensure coherence and reliability across all analyses.

All statistical analyses were performed using RStudio version 4.3.1. To estimate causal effects, we utilized the “TwoSampleMR” package, while the detection of horizontal pleiotropy and outliers were conducted using the “MR-PRESSO” package (Hemani et al., 2018; Verbanck et al., 2018). MVMR analysis was performed with the “MVMR” and “Mendelian Randomization” packages. The significance cutoff was set at *p* < 0.05, and the resulting MR estimates are presented as odds ratios (ORs) along with their corresponding 95% confidence intervals (CIs).

## 3 Results

### 3.1 Two-sample MR of gut microbiotas (exposure) on anxiety disorders (outcome)

Initially, 1,579 SNPs were selected as IVs for 119 bacterial genera after excluding unknown bacterial genera from the GWAS statistics (as shown in Supplementary Table S2). These IVs were then used in the forward MR analysis to investigate the potential causal effect of bacterial genera on anxiety disorders. The results of the initial MR analysis are presented in Supplementary Table S3. As shown in Table 1, the IVW estimate suggested that the genus *Eubacterium nodatum group* [OR = 0.902, 95%CI: 0.837–0.972, *p* = 0.007], and genus *Ruminococcaceae UCG011* [OR = 0.905, 95%CI: 0.831–0.987, *p* = 0.023] protected against anxiety disorders. However, the genus *Lachnospiraceae UCG010* [OR = 1.251, 95%CI: 1.028–1.522, *p* = 0.025] was associated with an increased risk of anxiety disorders.

**Table 1:** MR estimates and heterogeneity test for gut microbiota (exposure) on anxiety disorders (outcome).

Bacterial genera (exposure)	MR method	No. of SNP	Beta	SE	OR	95% CI	*p*-value	Heterogeneity test		
								Cochran’s Q	Q_df	*p*-value
*Eubacterium nodatum group*	IVW	11	−0.103	0.038	0.902	0.837–0.972	0.007	6.018	10	0.814
	MR-Egger	11	−0.085	0.170	0.918	0.658–1.281	0.628	6.006	9	0.739
	WME	11	−0.100	0.052	0.905	0.817–1.002	0.055	–	–	–
	Weighted model	11	−0.101	0.079	0.904	0.775–1.056	0.232	–	–	–
	Simple model	11	−0.112	0.093	0.894	0.746–1.073	0.257	–	–	–
*Ruminococcaceae UCG011*	IVW	8	−0.099	0.044	0.905	0.831–0.987	0.023	6.154	7	0.522
	MR-Egger	8	−0.307	0.219	0.735	0.478–1.130	0.210	5.217	6	0.516
	WME	8	−0.040	0.059	0.961	0.857–1.078	0.494	–	–	–
	Weighted model	8	−0.014	0.101	0.986	0.808–1.202	0.890	–	–	–
	Simple model	8	−0.014	0.097	0.986	0.816–1.191	0.885	–	–	–
*Lachnospiraceae UCG010*	IVW	10	0.224	0.100	1.251	1.028–1.522	0.025	14.094	9	0.119
	MR-Egger	10	−0.008	0.312	0.992	0.538–1.827	0.979	13.077	8	0.109
	WME	10	0.233	0.115	1.262	1.007–1.582	0.044	–	–	–
	Weighted model	10	0.251	0.167	1.285	0.926–1.783	0.167	–	–	–
	Simple model	10	0.226	0.172	1.253	0.895–1.754	0.221	–	–	–

MR, Mendelian randomization; SNP, single nucleotide polymorphism; SE, standard error; OR, odds ratio; CI, confidence interval; IVW, inverse variance weighted; WME, weighted median estimator.

**Figure 2:**
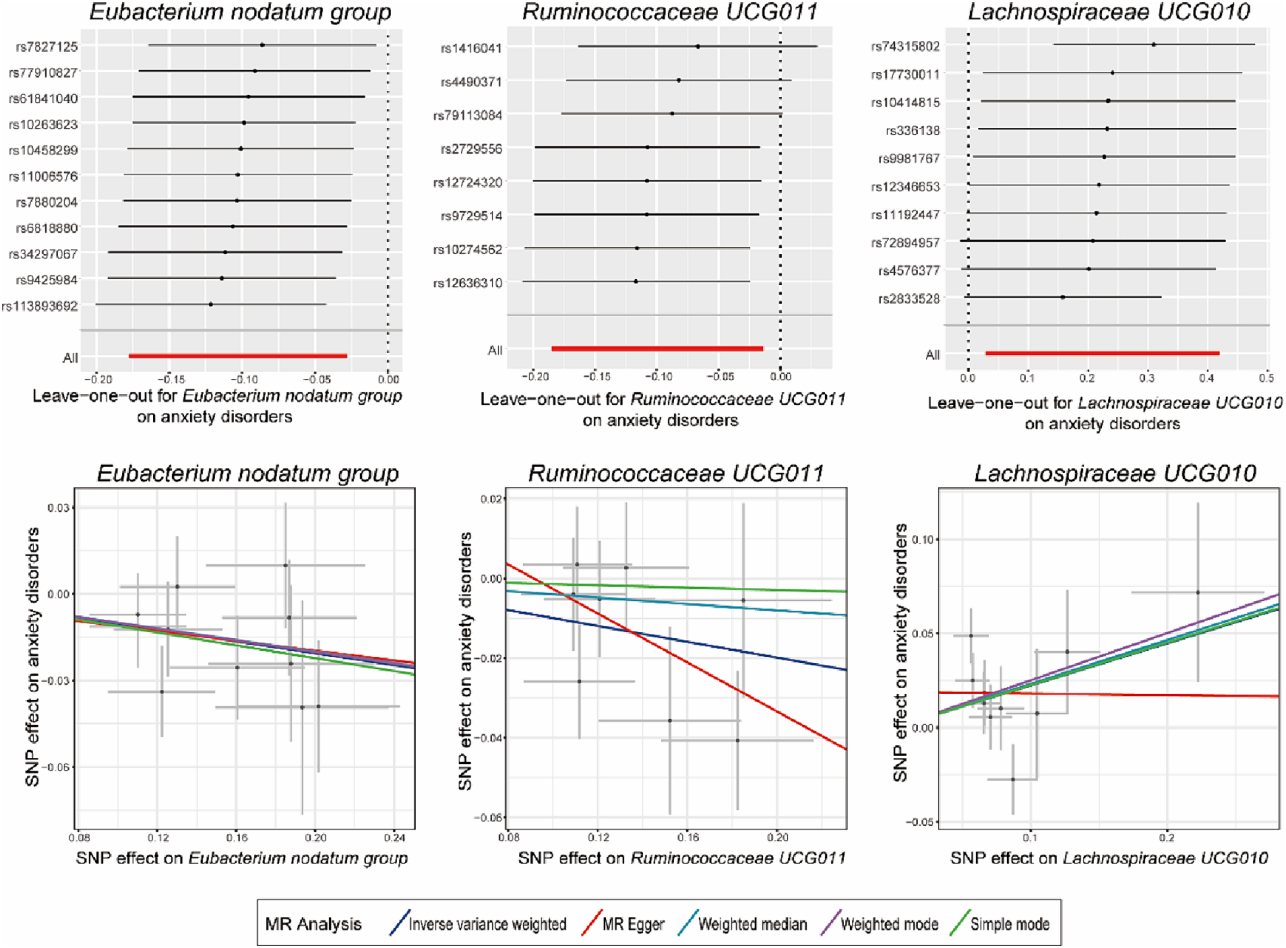
Leave-one-out plots and scatter plots for the causal effects of identified bacterial genera on the anxiety disorders. MR, Mendelian randomization; SNP, single nucleotide polymorphism; MR Egger, MR-Egger regression.

As presented in Tables 1, 2, Cochran’s Q statistic for both the MR-IVW and MR-Egger regression analyses did not show significant IV heterogeneity (*p* > 0.05 for all), indicating that heterogeneity bias was unlikely to have influenced our results. Furthermore, the results of the horizontal pleiotropy tests, including the MR-Egger intercept test and the MR-PRESSO global test, also showed no significant evidence of horizontal pleiotropy (*p* > 0.05 for all). These findings were supported by the leave-one-out plots and scatter plots shown in Figure 2.

**Table 2:** MR-Egger intercept test and MR-PRESSO analysis for associations between gut microbiota (exposure) and anxiety disorders (outcome).

Bacterial genera (exposure)	MR-Egger intercept test		MR-PRESSO analysis					
	Egger_ intercept	*p*-value	Causal estimate	SD	*T*	*p*-value	RSSobs	Global test *p*-value
*Eubacterium nodatum group*	−0.003	0.917	−0.103	0.030	−3.475	0.006	7.369	0.831
*Ruminococcaceae UCG011*	0.028	0.370	−0.099	0.041	−2.418	0.046	8.353	0.520
*Lachnospiraceae UCG010*	0.019	0.453	0.224	0.100	2.238	0.052	17.978	0.123

MR-PRESSO, Mendelian randomization pleiotropy RESidual sum and outlier; SD, standard deviation; RSSobs, observed residual sum of squares.

### 3.2 Reverse MR analysis of anxiety disorders (exposure) on identified bacterial genera (outcome)

Reverse MR analysis was next performed to evaluate the causal effect of anxiety disorders on the three identified bacterial genera belonging to the genus *Eubacterium nodatum group*, genus *Ruminococcaceae UCG011*, and genus *Lachnospiraceae UCG010*. After selecting suitable SNPs and removing palindromic ones in Supplementary Table S4, we used 13, 14, and 15 SNPs, respectively, as potential IVs in the reverse MR analysis to investigate the potential causal effect of anxiety disorders on the following bacterial genera: *Eubacterium nodatum group*, *Ruminococcaceae UCG011*, and *Lachnospiraceae UCG010*. As shown in Table 3, reverse MR analysis showed that there was no evidence of a causal effect of anxiety disorders on these three identified bacterial genera (*p* > 0.05 for all). As presented in Tables 3, 4, Cochran’s Q statistic showed that there was no significant heterogeneity of the IVs (*p* > 0.05 for all), and the results of the MR-Egger intercept test and MR-PRESSO global test also did not detect significant horizontal pleiotropy (*p* > 0.05 for all).

**Table 3:** Reverse MR estimates and heterogeneity test for anxiety disorders (exposure) on identified bacterial genera (outcome).

Bacterial genera (outcome)	MR method	No. of SNP	Beta	SE	OR	95% CI	*p*-value	Heterogeneity test		
								Cochran’s Q	Q_df	*p*-value
*Eubacterium nodatum group*	IVW	13	0.051	0.123	1.052	0.827–1.340	0.679	19.344	12	0.081
	MR-Egger	13	−0.319	0.334	0.727	0.378–1.399	0.360	17.143	11	0.104
	WME	13	0.007	0.146	1.007	0.756–1.341	0.963	–	–	–
	Weighted model	13	−0.025	0.206	0.975	0.651–1.461	0.904	–	–	–
	Simple model	13	0.112	0.237	1.119	0.702–1.781	0.645	–	–	–
*Ruminococcaceae UCG011*	IVW	14	0.017	0.090	1.017	0.853–1.212	0.854	6.374	13	0.931
	MR-Egger	14	−0.040	0.257	0.961	0.581–1.590	0.878	6.318	12	0.899
	WME	14	0.011	0.123	1.011	0.794–1.287	0.931	–	–	–
	Weighted model	14	−0.024	0.193	0.976	0.669–1.424	0.902	–	–	–
	Simple model	14	−0.175	0.204	0.839	0.563–1.252	0.406	–	–	–
*Lachnospiraceae UCG010*	IVW	15	−0.021	0.052	0.979	0.884–1.083	0.679	16.984	14	0.257
	MR-Egger	15	0.098	0.150	1.103	0.822–1.481	0.523	16.086	13	0.245
	WME	15	−0.021	0.068	0.979	0.856–1.119	0.755	–	–	–
	Weighted model	15	0.075	0.110	1.078	0.868–1.338	0.508	–	–	–
	Simple model	15	−0.062	0.123	0.940	0.739–1.196	0.622	–	–	–

MR, Mendelian randomization; SNP, single nucleotide polymorphism; SE, standard error; OR, odds ratio; CI, confidence interval; IVW, inverse variance weighted; WME, weighted median estimator.

**Table 4:** MR-Egger intercept test and MR-PRESSO analysis for associations between anxiety disorders (exposure) and identified bacterial genera (outcome).

Bacterial genera (exposure)	MR-Egger intercept test		MR-PRESSO analysis					
	Egger_intercept	*p*-value	Causal estimate	SD	*T*	*p*-value	RSSobs	Global test *p*-value
*Eubacterium nodatum group*	0.036	0.260	0.051	0.123	0.413	0.687	22.516	0.100
*Ruminococcaceae UCG011*	0.005	0.818	0.017	0.063	0.263	0.797	7.329	0.938
*Lachnospiraceae UCG010*	−0.011	0.410	−0.021	0.052	−0.414	0.685	19.408	0.283

MR-PRESSO, Mendelian randomization pleiotropy RESidual sum and outlier; SD, standard deviation; RSSobs, observed residual sum of squares.

### 3.3 MVMR to assess the effect of neurotransmitters associated metabolites on observed associations between identified bacterial genera (exposure) on anxiety disorders (outcome)

Considering the possible contribution of neurotransmitter-associated metabolites to the pathobiology of gut microbiota-associated anxiety disorders, we used MVMR for the observed significant associations (the results in Table 1) adjusted for seven common metabolites (tryptophan, tyrosine, phenylalanine, glutamate, glycine, cortisol and isovalerate). The MVMR results are reported in Table 5. When employing the MVMR-IVW method to account for the combined influence of all seven metabolites, the previously identified significant associations between identified bacterial genera and anxiety disorders were no longer significant following adjustments (*p* > 0.05 for all), and there was no significant heterogeneity in these results (*p* > 0.05 for all). Subsequently, focusing on the genus *Eubacterium nodatum group*, genus *Ruminococcaceae UCG011*, and genus *Lachnospiraceae UCG010*, we next adjusted only one metabolite at a time (Table 6). Signals of a decrease in significance of the association were detected by MVMR-IVW for genus *Eubacterium nodatum group* [*p* = 0.931 for tryptophan; *p* = 0.268 for tyrosine; *p* = 0.151 for phenylalanine; *p* = 0.083 for glycine; *p* = 0.127 for cortisol], genus *Ruminococcaceae UCG011* [*p* = 0.346 for tyrosine; *p* = 0.124 for phenylalanine; *p* = 0.088 for glycine; *p* = 0.063 for cortisol], and genus *Lachnospiraceae UCG010* [*p* = 0.149 for tryptophan]. Additionally, heterogeneity testing indicated that our MVMR estimates were unlikely biased by heterogeneity (*p* > 0.05 for all).

**Table 5:** MVMR results of causal links between identified bacterial genera (exposure) on anxiety disorders (outcome) after adjusting for all neurotransmitter-associated metabolites.

Bacterial genera (exposure)	Adjustment of confounder	MR method	Beta	SE	OR	95% CI	*p*-value	Heterogeneity *p*-value
*Eubacterium nodatum group*	Seven metabolites	MVMR-IVW	0.020	0.040	1.020	0.944–1.102	0.621	0.166
		MVMR-Egger	−0.038	0.056	0.963	0.863–1.074	0.500	0.182
*Ruminococcaceae UCG011*	Seven metabolites	MVMR-IVW	−0.075	0.040	0.928	0.858–1.002	0.057	0.213
		MVMR-Egger	−0.078	0.058	0.924	0.825–1.036	0.176	0.197
*Lachnospiraceae UCG010*	Seven metabolites	MVMR-IVW	0.083	0.082	1.087	0.925–1.277	0.312	0.185
		MVMR-Egger	0.316	0.111	1.372	1.104–1.704	0.004	0.328

MVMR, multivariable Mendelian randomization; SE, standard error; OR, odds ratio; CI, confidence interval; IVW, inverse variance weighted.

**Table 6:** Multivariable MR results of causal links between identified bacterial genera (exposure) on anxiety disorders (outcome) after adjusting for specific neurotransmitter-associated metabolite.

Bacterial genera (exposure)	Adjustment of confounder	MR method	Beta	SE	OR	95% CI	*p*-value	Heterogeneity *p*-value
*Eubacterium nodatum group*	Tryptophan	MVMR-IVW	0.004	0.042	1.004	0.924–1.090	0.931	0.186
		MVMR-Egger	−0.031	0.059	0.969	0.864–1.088	0.595	0.182
	Tyrosine	MVMR-IVW	−0.050	0.045	0.951	0.872–1.039	0.268	0.763
		MVMR-Egger	−0.120	0.060	0.887	0.788–0.998	0.046	0.850
	Phenylalanine	MVMR-IVW	−0.075	0.052	0.928	0.838–1.028	0.151	0.325
		MVMR-Egger	−0.234	0.089	0.792	0.665–0.942	0.009	0.701
	Glutamate	MVMR-IVW	−0.098	0.049	0.907	0.823–0.998	0.046	0.955
		MVMR-Egger	−0.151	0.220	0.860	0.559–1.323	0.492	0.917
	Glycine	MVMR-IVW	−0.082	0.047	0.921	0.840–1.011	0.083	0.812
		MVMR-Egger	−0.096	0.060	0.908	0.808–1.022	0.109	0.775
	Cortisol	MVMR-IVW	−0.074	0.048	0.928	0.845–1.021	0.127	0.784
		MVMR-Egger	−0.195	0.074	0.822	0.711–0.952	0.009	0.984
	Isovalerate	MVMR-IVW	−0.109	0.049	0.897	0.815–0.986	0.025	0.851
		MVMR-Egger	−0.160	0.084	0.852	0.723–1.004	0.056	0.835
*Ruminococcaceae UCG011*	Tryptophan	MVMR-IVW	−0.083	0.042	0.920	0.847–0.999	0.047	0.232
		MVMR-Egger	−0.091	0.061	0.913	0.810–1.028	0.132	0.216
	Tyrosine	MVMR-IVW	−0.045	0.047	0.956	0.872–1.049	0.346	0.787
		MVMR-Egger	−0.079	0.067	0.924	0.811–1.053	0.235	0.772
	Phenylalanine	MVMR-IVW	−0.089	0.058	0.915	0.817–1.025	0.124	0.131
		MVMR-Egger	−0.113	0.116	0.893	0.711–1.120	0.328	0.089
	Glutamate	MVMR-IVW	−0.091	0.045	0.913	0.835–0.998	0.045	0.572
		MVMR-Egger	−0.066	0.105	0.936	0.762–1.150	0.529	0.463
	Glycine	MVMR-IVW	−0.074	0.043	0.929	0.853–1.011	0.088	0.655
		MVMR-Egger	−0.069	0.060	0.933	0.829–1.050	0.251	0.599
	Cortisol	MVMR-IVW	−0.084	0.045	0.919	0.841–1.005	0.063	0.536
		MVMR-Egger	−0.191	0.074	0.826	0.714–0.955	0.010	0.761
	Isovalerate	MVMR-IVW	−0.113	0.045	0.893	0.817–0.976	0.013	0.725
		MVMR-Egge	−0.055	0.091	0.946	0.792–1.131	0.543	0.690
*Lachnospiraceae UCG010*	Tryptophan	MVMR-IVW	0.120	0.083	1.128	0.958–1.327	0.149	0.202
		MVMR-Egger	0.317	0.108	1.373	1.110–1.697	0.003	0.329
	Tyrosine	MVMR-IVW	0.290	0.086	1.336	1.130–1.580	0.001	0.662
		MVMR-Egger	0.377	0.108	1.458	1.180–1.802	0.001	0.700
	Phenylalanine	MVMR-IVW	0.294	0.113	1.342	1.075–1.675	0.009	0.129
		MVMR-Egger	0.274	0.202	1.315	0.886–1.953	0.175	0.088
	Glutamate	MVMR-IVW	0.309	0.091	1.363	1.140–1.629	0.001	0.450
		MVMR-Egger	0.363	0.158	1.438	1.055–1.958	0.021	0.366
	Glycine	MVMR-IVW	0.278	0.087	1.321	1.115–1.565	0.001	0.601
		MVMR-Egger	0.429	0.112	1.536	1.234–1.912	0.001	0.799
	Cortisol	MVMR-IVW	0.287	0.103	1.332	1.089–1.630	0.005	0.321
		MVMR-Egge	0.377	0.141	1.458	1.105–1.923	0.008	0.316
	Isovalerate	MVMR-IVW	0.319	0.092	1.376	1.149–1.648	0.001	0.421
		MVMR-Egge	0.449	0.137	1.566	1.196–2.051	0.001	0.470

MVMR, multivariable Mendelian randomization; SE, standard error; OR, odds ratio; CI, confidence interval; IVW, inverse variance weighted.

### 3.4 MVMR to assess the impact of dietary patterns, drug or alcohol consumption, and lifestyle factors on observed associations between the identified bacterial genera (exposure) and anxiety disorders (outcome)

Considering the potential impact of dietary patterns, drug or alcohol consumption, and lifestyle factors on the causal effects of the identified bacterial genera on anxiety disorders, we used MVMR for observed significant associations (the results in Table 1) adjusted for dietary patterns, drug or alcohol consumption, and lifestyle factors. We implemented adjustments for dietary pattern (salt added to food, processed meat intake, intake of sugar added to cereal), drug or alcohol consumption (more than once), and lifestyle factors (smoking status and physical activity) one at a time, and the MVMR results are reported in Table 7. Signals of a decline in the significance of association were detected in the MVMR-IVW for genus *Eubacterium nodatum group* [*p* = 0.223 for salt added to food; *p* = 0.646 for processed meat intake; *p* = 0.224 for smoking status], genus *Ruminococcaceae UCG011* [*p* = 0.418 for salt added to food; *p* = 0.951 for processed meat intake; *p* = 0.748 for smoking status; *p* = 0.083 for physical activity], and genus *Lachnospiraceae UCG010* [*p* = 0.729 for salt added to food; *p* = 0.082 for processed meat intake; *p* = 0.107 for intake of sugar added to cereal; *p* = 0.844 for smoking status; *p* = 0.054 for physical activity]. Heterogeneity testing indicated that our MVMR estimates were unlikely biased by heterogeneity (most *p* > 0.05).

**Table 7:** MVMR results of causal links between the identified bacterial genera (exposure) and anxiety disorders (outcome) after adjusting for dietary pattern, drug or alcohol consumption, and lifestyle factors.

Bacterial genera (exposure)	Adjustment of confounder	MR method	Beta	SE	OR	95% CI	*p*-value	Heterogeneity *p*-value
*Eubacterium nodatum group*	Salt added to food	MVMR-IVW	−0.042	0.034	0.959	0.897–1.026	0.223	0.049
		MVMR-Egger	−0.068	0.050	0.934	0.846–1.031	0.175	0.047
	Processed meat intake	MVMR-IVW	−0.022	0.048	0.978	0.891–1.074	0.646	5e-04
		MVMR-Egger	−0.085	0.068	0.918	0.804–1.049	0.209	6e-04
	Intake of sugar added to cereal	MVMR-IVW	−0.109	0.046	0.897	0.820–0.981	0.017	0.125
		MVMR-Egger	−0.183	0.070	0.832	0.725–0.955	0.009	0.164
	Drug or alcohol consumption	MVMR-IVW	−0.100	0.038	0.905	0.840–0.973	0.008	0.684
		MVMR-Egger	−0.040	0.059	0.961	0.857–1.078	0.500	0.739
	Smoking status	MVMR-IVW	−0.037	0.030	0.964	0.909–1.023	0.224	0.044
		MVMR-Egger	−0.033	0.047	0.967	0.883–1.060	0.480	0.041
	Physical activity	MVMR-IVW	−0.095	0.036	0.909	0.848–0.975	0.007	0.900
		MVMR-Egger	−0.139	0.047	0.871	0.793–0.955	0.004	0.921
*Ruminococcaceae UCG011*	Salt added to food	MVMR-IVW	−0.029	0.036	0.971	0.905–1.042	0.418	0.047
		MVMR-Egger	−0.028	0.054	0.973	0.874–1.082	0.611	0.043
	Processed meat intake	MVMR-IVW	−0.003	0.048	0.997	0.908–1.095	0.951	5e-04
		MVMR-Egger	0.001	0.064	1.001	0.884–1.133	0.992	4e-04
	Intake of sugar added to cereal	MVMR-IVW	−0.111	0.052	0.895	0.809–0.990	0.031	0.100
		MVMR-Egger	−0.113	0.085	0.893	0.756–1.055	0.183	0.074
	Drug or alcohol consumption	MVMR-IVW	−0.097	0.042	0.907	0.835–0.986	0.022	0.656
		MVMR-Egger	−0.058	0.069	0.944	0.825–1.080	0.401	0.628
	Smoking status	MVMR-IVW	−0.010	0.032	0.990	0.930–1.054	0.748	0.068
		MVMR-Egger	−0.053	0.049	0.948	0.861–1.045	0.282	0.070
	Physical activity	MVMR-IVW	−0.071	0.041	0.931	0.859–1.009	0.083	0.477
		MVMR-Egger	−0.114	0.059	0.892	0.795–1.001	0.052	0.479
*Lachnospiraceae UCG010*	Salt added to food	MVMR-IVW	−0.024	0.070	0.976	0.851–1.120	0.729	0.050
		MVMR-Egger	0.104	0.099	1.109	0.913–1.347	0.296	0.063
	Processed meat intake	MVMR-IVW	0.150	0.086	1.162	0.981–1.377	0.082	8e-04
		MVMR-Egger	0.053	0.11	1.054	0.837–1.327	0.654	9e-04
	Intake of sugar added to cereal	MVMR-IVW	0.177	0.110	1.194	0.962–1.482	0.107	0.088
		MVMR-Egger	0.038	0.145	1.039	0.782–1.381	0.792	0.129
	Drug or alcohol consumption	MVMR-IVW	0.230	0.085	1.259	1.065–1.489	0.007	0.220
		MVMR-Egger	0.170	0.127	1.186	0.925–1.520	0.180	0.198
	Smoking status	MVMR-IVW	0.013	0.064	1.013	0.893–1.148	0.844	0.070
		MVMR-Egger	0.042	0.095	1.043	0.866–1.257	0.656	0.066
	Physical activity	MVMR-IVW	0.161	0.083	1.174	0.997–1.383	0.054	0.231
		MVMR-Egger	0.141	0.103	1.151	0.941–1.409	0.171	0.205

MVMR, multivariable Mendelian randomization; SE, standard error; OR, odds ratio; CI, confidence interval; IVW, inverse variance weighted.

## 4 Discussion

This study exploited the summary statistics of gut microbiota obtained from the largest GWAS meta-analysis conducted by the MiBioGen consortium, which included 18,340 individuals across 24 separate cohorts. Predicted through genetic factors at a more precise genus level (utilizing a total of 1,579 SNPs for 119 bacterial genera, after excluding unknown bacterial genera), our MR study provides evidence that gut microbiota can have a causal effect on anxiety disorders. Specifically, at the genus level, *Eubacterium nodatum group* and *Ruminococcaceae UCG011* appeared to be protective, while *Ruminococcaceae UCG010* was associated with an increased risk of anxiety disorders. Reverse MR analysis and a series of sensitivity analyses also confirmed the robustness of these findings. Currently, research on the association between anxiety disorders and these identified bacterial genera are lacking. However, interventions targeting improvements in the structural composition of the gut microbiota, such as the abundance of *Eubacterium nodatum group* genus, have shown promising efficacy in the treatment of attention deficit hyperactivity disorder (Tang et al., 2022). Another study provided further evidence that remodeling the gut microbiota by restoring dysbiosis characterized by the presence of genus *Eubacterium nodatum group* and other relevant bacterial genera effectively improved the disruption of the gut-brain barrier, thereby providing a potential therapeutic approach for the treatment of cerebral ischemic stroke (Chen et al., 2019). The genus *Ruminococcaceae UCG011* and genus *Ruminococcaceae UCG010*, belonging to family *Ruminococcaceae*, have been reported as associated with some diseases such as leukemia (Chen et al., 2023), type 2 diabetes (Li and Li, 2023), and esophageal cancer (Wang et al., 2024), but they have yet to be studies in the context of mental health disorders. However, multiple clinical studies have demonstrated that psychological distress and mental illness, such as depression and anxiety, are associated with alterations in the abundance of *Ruminococcaceae* family members (Chen et al., 2021; Chin Fatt et al., 2023), and animal studies have suggested that regulating the abundance of *Ruminococcaceae* family members with various interventions can promote the production of SCFAs and other neurochemical-related metabolites. These metabolites, in turn, modulate neurotransmitter-associated metabolism, inflammation, and the microbiota-gut-brain axis, thereby alleviating depressive and anxiety-like behaviors in different mouse models of chronic stress (Chen Y. et al., 2022; Zhou et al., 2022).

Given the limited research on the bacterial genera associated with anxiety disorders identified in our MR analysis, it is crucial to investigate the role of metabolites that are closely linked to gut microbiota and anxiety disorders. Our study utilized large-scale GWAS data and involved MVMR analysis to identify potential metabolic pathways involving neurotransmitter-associated metabolites, which are believed to facilitate communication between the gut microbiota and the brain. Our results showed that tyrosine, phenylalanine, glycine, and cortisol played a crucial role in the protective effects of genus *Eubacterium nodatum group* and genus *Ruminococcaceae UCG011* on anxiety disorders. First, tyrosine is the precursor to dopamine, a neurotransmitter responsible for regulating brain functions related to emotions, rewards, and motivation, and the gut microbiota has been found to regulate tyrosine metabolism, which subsequently affects dopamine synthesis and release to the CNS. Imbalances in the dopamine system can interfere with brain’s limbic system activities, resulting in exaggerated neuronal excitations in anxiety-related brain areas such as the amygdala and prefrontal cortex, leading to emotional instability and worsening anxiety disorders (Needham et al., 2022; Zhang D. et al., 2022). Second, phenylalanine not only serves as a precursor of tyrosine but also plays a crucial role in promoting the synthesis and secretion of endorphins in the brain. Indeed, recent studies have shown that the gut microbiota can help with the digestion and absorption of phenylalanine, and the metabolic activity of phenylalanine can be affected by an imbalanced gut microbiota, in turn affecting the synthesis and secretion of endorphins in the CNS, ultimately influencing the occurrence and severity of anxiety disorders (Deng et al., 2022; Zhang D.-D. et al., 2022). Third, glycine can effectively suppress neuronal activity and impact brain excitability. Several studies have shown that the gut microbiota can disrupt glycine metabolism and glycine receptor signaling, thereby increasing the risk of anxiety and other neuropsychiatric disorders (Lin et al., 2021; Zhang D. et al., 2022). Lastly, cortisol, a hormone secreted by the adrenal gland, plays a crucial role in stress responses. The gut microbiota affects cortisol synthesis and metabolism through the breakdown of fibers and polysaccharides to produce metabolites like SCFAs. Sustained elevation of cortisol levels not only negatively affects the hippocampus and prefrontal cortex of the brain, impairing mood regulation, decision-making, and cognitive functions through activation of microglia-induced neuroinflammation, but also alters neurotransmitter systems in the brain, such as the serotonin and GABA systems critical for emotional regulation (Dalile et al., 2020; Tian et al., 2023). In addition, only tryptophan was found to have a potential impact on the negative effect of genus *Ruminococcaceae UCG010* on anxiety disorders. Tryptophan is an essential amino acid for the synthesis of serotonin, which plays critical roles in regulating mood, behavior, and cognition. It has been shown that disruptions in the gut microbiota can have consequences on tryptophan metabolism and utilization by (1) giving rise to the production of tryptophan antagonists, and (2) regulating tryptophan transporters to reduce tryptophan reaching the brain. These processes can result in aberrant function and reduced levels of CNS serotonin, contributing to the development and continuation of anxiety disorders (Deng et al., 2021; Jia et al., 2024).

Previous studies have suggested that the gut microbiota may be affected by dietary patterns such as salt added to food, processed meat intake, and intake of sugar added to cereals; drug or alcohol consumption; and lifestyle factors such as smoking and physical activity (Zhu et al., 2020; Simpson et al., 2021; Cataldi et al., 2022). We therefore used MVMR analysis to assess the impact of these confounding variables on the observed causal associations between the identified bacterial genera and anxiety disorders. Our findings showed that excessive consumption of processed meat products and sugar and improper intake of salt (too much or too little) increased the risk of developing anxiety by regulating the gut microbiota. Regarding dietary patterns, emerging evidence suggests that unhealthy dietary can alter the gut microbiota composition, in turn disrupting metabolic functions and the immune system, thereby inducing anxiety disorders (Bear et al., 2020; Zhu et al., 2020). Additionally, we also found that controlling smoking and engaging in moderate physical activity are important lifestyle factors that reduce the risk of anxiety disorders through interactions with the identified bacterial genera. Indeed, recent studies have reported that tobacco smoke components such as nicotine are present in the gastrointestinal tract and disrupt the gut microbiota (Chen et al., 2024), interfering with vagal nerve signaling and neurotransmission, as well as inducing CNS inflammation, ultimately increasing the risk of mental health illnesses such as depression, schizophrenia, and anxiety disorders (Curtis et al., 2019; Wang et al., 2023). Furthermore, prioritizing aerobic physical activity at moderate intensity, rather than prolonged intensity, seems to be useful in regulating the diversity and richness of beneficial bacterial taxa in both humans and animals. This improves physical performance and triggers beneficial changes in the brain, including an increase in the production of neurotransmitters such as dopamine, glutamate, and GABA and reducing central inflammatory responses (Cataldi et al., 2022). Therefore, with a growing understanding of the mechanisms involved in the complex interplay between dietary and lifestyle factors, the gut microbiota, and its impact on mental illness, specific dietary and lifestyle patterns might potentially be translated into clinical practice to help prevent anxiety and mood disorders. We propose that dietary and lifestyle interventions have the dual benefit of a direct impact on gut and brain physiology and an indirect effect via the gut microbiota, and the incorporation of dietary and lifestyle interventions may prove to be an attractive and cost-effective alternative or adjuvant therapy for the clinical management of anxiety disorders.

The main strength of this study is the use of bidirectional MR analysis to investigate the causal effects and identify specific bacterial taxa at the genus level linked to anxiety disorders. This approach effectively minimizes potential confounding factors and reverse causation, providing reliable evidence for causal relationships between gut microbiotas and anxiety disorders. The use of multiple statistical models and sensitivity analyses ensured the accuracy and validity of the estimated causal effects. Moreover, applying MVMR analysis to explore the potential impact of various neurotransmitter-associated metabolites, which are highly associated with anxiety disorders, on the causal effects of identified bacterial genera on anxiety disorders might enable us to target metabolic pathways connecting the gut microbiota to anxiety disorders. Our study design allowed us to gain a deeper understanding of the complex interactions and gut-brain communication mechanisms mediating the gut microbiota and anxiety disorders. Building on this, we conducted additional MVMR analyses to investigate the impact of relevant dietary patterns, drug or alcohol use, and lifestyle factors on the causal effects of specific bacteria genera on anxiety disorders. By modifying dietary patterns and adjusting lifestyle factors such as smoking and physical activity, it may be possible to develop more precise and effective strategies for targeting specific gut microbiota to manage anxiety disorders.

However, our study has some limitations. First, our study only focused on individuals of European ancestry and did not consider the impact of sex. Thus, caution is needed when generalizing our findings to other populations, and future research should include diverse populations and conduct sex-specific analyses. Second, it is essential to study the gut microbiome as a comprehensive ecosystem, rather than focusing solely on individual species or strains. Longitudinal studies would be valuable in revealing the dynamic changes in gut microbiota associated with the development of anxiety disorders. Third, in our study, we analyzed metabolites in multivariable MR that were detected in human serum. While we acknowledge that fecal samples may provide more appropriate and direct information, unfortunately, such data are currently unavailable. Additionally, other neurotransmitters, such as norepinephrine, and more crucial SCFAs like propionate and butyrate, might be important to consider, but summary data on these compounds or their related metabolites were also lacking. In summary, although our research provides valuable insights into the relationship between gut microbiota and anxiety disorders, it is crucial to acknowledge and address these limitations through further high-quality experimental and clinical studies. This should involve exploring higher taxonomic levels, incorporating the broader microbial diversity within the gut ecosystem, and considering the broader confounding factors within dietary and lifestyle variables.

## 5 Conclusion

Overall, we detected three causal associations through MR analysis on the impacts of 119 bacterial taxa on anxiety disorders. Among them, the genus *Eubacterium nodatum group* and genus *Ruminococcaceae UCG011* protected against anxiety disorders, while genus *Ruminococcaceae UCG011* was associated with an increased risk of anxiety disorders. A metabolite-dependent mechanism, primarily driven by tyrosine, phenylalanine, glycine, and cortisol, played a crucial role in genus *Eubacterium nodatum group* and genus *Ruminococcaceae UCG011* on anxiety disorders, while tryptophan was found to have a potential impact on genus *Ruminococcaceae UCG011*. In addition, excessive consumption of processed meat products and sugar, improper salt intake, smoking status, and excessive and prolonged physical activity could increase the risk of developing anxiety by regulating the gut microbiota. Further research is necessary to advance our understanding of the biological mechanisms responsible for the causal relationship between the microbiota-gut-brain axis and onset and development of anxiety disorders. This could help in the discovery of valuable biomarkers and potential therapy targets.

## Data availability statement

The original contributions presented in the study are included in the article/Supplementary material, further inquiries can be directed to the corresponding authors.

## Author contributions

M-MX: Conceptualization, Data curation, Formal analysis, Funding acquisition, Investigation, Methodology, Project administration, Resources, Software, Supervision, Validation, Visualization, Writing – original draft, Writing – review & editing. W-HQ: Supervision, Data curation, Formal analysis, Investigation, Methodology, Project administration, Software, Validation, Visualization, Writing – original draft. Q-YM: Resources, Data curation, Formal analysis, Investigation, Methodology, Project administration, Software, Supervision, Validation, Writing – original draft. Z-YY: Project administration, Validation, Writing – review & editing, Data curation, Formal analysis, Investigation, Methodology, Software, Supervision. W-MY: Writing – review & editing, Conceptualization, Data curation, Investigation, Methodology, Project administration, Software, Supervision, Validation, Visualization. T-NH: Supervision, Writing – review & editing, Data curation, Formal analysis, Methodology. YG: Conceptualization, Data curation, Formal analysis, Funding acquisition, Investigation, Methodology, Project administration, Resources, Software, Supervision, Validation, Visualization, Writing – review & editing, Writing – original draft. X-YC: Writing – original draft, Conceptualization, Data curation, Formal analysis, Funding acquisition, Investigation, Methodology, Project administration, Resources, Software, Supervision, Validation, Visualization, Writing – review & editing.

## Footnotes

^1^ https://metabolomics.helmholtz-muenchen.de/gwas/

^2^ http://www.phenoscanner.medschl.cam.ac.uk/
